# Effect of (−)-Epicatechin on Mitochondrial Homeostasis in Skeletal Muscle of Female Obese Rats

**DOI:** 10.3390/molecules31061050

**Published:** 2026-03-22

**Authors:** Elena de la C. Herrera-Cogco, Socorro Herrera-Meza, Yuridia Martínez-Meza, Javier Pérez-Durán, Guillermo Ceballos, Enrique Méndez-Bolaina, Nayelli Nájera

**Affiliations:** 1 Facultad de Ciencias Químicas, Universidad Veracruzana, Prolongación de Oriente 6, No. 1009, Orizaba 94340, Veracruz, Mexico; elenahc.sosa@gmail.com; 2 Instituto de Investigaciones Psicológicas, Universidad Veracruzana, Xalapa, Lomas del Estadio s/n, Xalapa 91000, Veracruz, Mexico; 3 Escuela Superior de Medicina, Instituto Politécnico Nacional, Ciudad de México 11340, Mexico; 4 Reproductive and Perinatal Health Research Department, Instituto Nacional de Perinatología Isidro Espinosa de los Reyes, Ciudad de México 11000, Mexico

**Keywords:** epicatechin, sarcopenia, obesity, skeletal muscle, females

## Abstract

Background: Main risk factors associated with the development of sarcopenia (coexistence of muscle mass loss and dysfunction) are a sedentary lifestyle coupled with obesity. Associated mitochondrial dysfunction leads to energy deficits and perturbations in the balance between protein synthesis and degradation, thereby triggering muscle dysfunction or atrophy. Aside from exercise, which is challenging to implement and maintain, particularly in women, treatments for diminishing sarcopenia are scarce. The objective of the present study was to evaluate the effect of the flavanol (−)-epicatechin (EC) in a hypercaloric diet-induced obese female rat model. Muscle strength and endurance, as well as relative mitochondrial DNA content in skeletal muscle, were assessed. Methods: Female rats were fed a hypercaloric diet to induce obesity, as evidenced by increases in body weight, Lee index, and lipid profile alterations, and by abdominal fat accumulation, and to promote a sarcopenic phenotype. Functional tests of grip strength and mobility (treadmill) were performed. Mitochondrial relative content was evaluated by measuring the ratio of mtDNA/nuclear DNA, and the expression of genes related to mitochondrial biogenesis (*Pgc1-α*, *Tfam*), fusion (*Mfn1* and *Opa1*), fission (*Drp1* and *Fis1*), and mitophagy (*Pink1* and *Pkn*), and function; citrate synthase and *Ucp3* were also evaluated. Results: A significant decrease in mobility and strength was observed in obese female rats, accompanied by reduced mitochondrial numbers, activity, and dynamics, but not by changes in muscle size or weight. Treatment with EC induced mitochondrial biogenesis and positive changes in mitochondrial dynamics (fission and fusion) and activity, as measured indirectly by changes in citrate synthase and *Ucp3* expression. Discussion: Results reinforce the potential of EC as a modulator of mitochondrial function in dysfunctional conditions associated with obesity, thereby attenuating the mechanisms underlying sarcopenia.

## 1. Introduction

Sarcopenia is defined as the progressive loss of muscle mass, strength, and function [1]. One of the main risk factors for the development of sarcopenia is a sedentary lifestyle coupled with obesity (O). This becomes even more important due to the increasing prevalence of O worldwide, particularly in women [2].

In this context, the term “sarcopenic obesity” denotes the coexistence of sarcopenia (loss of muscle mass and function) and excess adipose tissue. Individuals with both conditions have a 24% higher risk of all-cause mortality [3]. Although obesity and sarcopenia are distinct conditions, they share altered molecular mechanisms that promote their development, forming a vicious circle [4].

Various molecular mechanisms contribute to the pathophysiology of sarcopenia, including altered proteostasis, satellite cell damage, and mitochondrial dysfunction [5].

In this regard, mitochondria play a crucial role in maintaining skeletal muscle health by supplying energy for contraction via ATP generated by oxidative phosphorylation. Therefore, mitochondrial dysfunction leads to energy deficits and perturbations in the balance between protein synthesis and degradation, thereby triggering muscle dysfunction or atrophy, primarily due to oxidative damage (increased reactive oxygen species production), dysregulation of mitochondrial dynamics, and impaired mitophagy [6,7].

Aside from exercise, treatments aimed at controlling sarcopenia are scarce, and exercise in O is challenging to implement and maintain, given the multiple associated pathologies. Several issues have been reported as limitations for implementing or maintaining exercise, such as a lack of motivation, anxiety or depression, or associated obesity fatigue [8]. Therefore, the search for alternatives to mitigate sarcopenia, independent of, but not excluding, exercise, remains an active area of research. In this regard, therapeutic alternatives using plant-derived molecules, such as polyphenols, have shown great potential; however, their systematic study remains limited, and further investigation of their effects could open new avenues for controlling sarcopenia.

Flavonoids are plant secondary metabolites found in cacao, tea, berry fruit, citrus, and other fruits and legumes. Flavonoid consumption, particularly (−)-epicatechin (EC), promotes vascular flow, improves mobility, and is associated with a decreased risk of age-associated sarcopenia [9].

Recently, we reported that an epicatechin-enriched product improves physical performance/mobility in subjects aged 60 and older, facilitating active engagement in physical activities and increasing mobility [10]. Based on these results, the objective of the present study was to evaluate the effect of a 4-week treatment with the natural flavanol EC in a diet-induced obese female rat model. Muscle strength and endurance, as well as relative mitochondrial DNA content in skeletal muscle, were assessed. In addition, to explore potential mechanisms underlying mitochondrial dynamics, the expression of genes involved in mitochondrial biogenesis and dynamics, as well as mitophagy, was assessed.

## 2. Results

### Effect of the HC Diet

**Figure 1:**
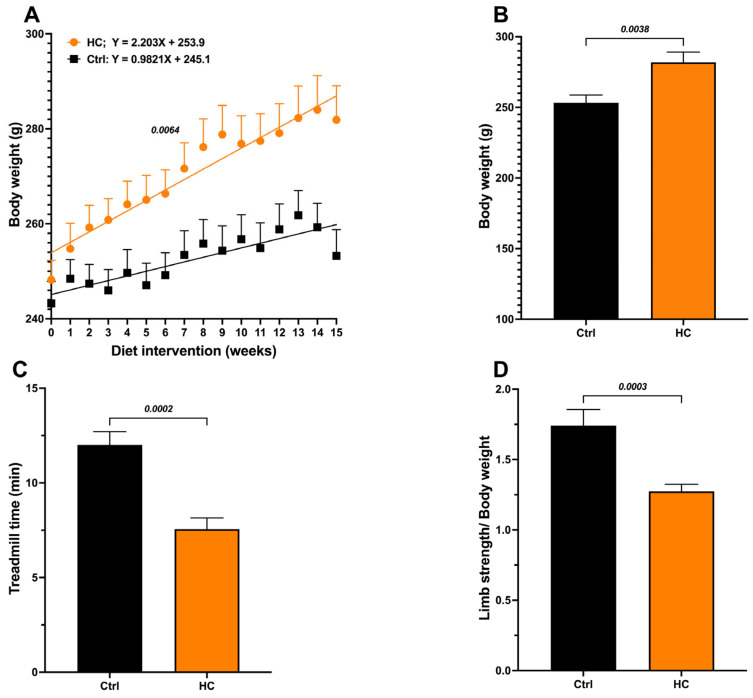
Effect of diet on body weight and functional test performance in female rats. (**A**) Body weight recorded weekly during the induction period with the HC diet. (**B**) Body weight at 15 weeks of diet. (**C**) Treadmill test at 15 weeks of diet and (**D**) Handgrip strength test at 15 weeks of diet. Data show mean ± SEM. Ctrl (*n* = 8) and HC diet (*n* = 16).

Animals fed the HC diet for 15 weeks showed a significant increase in body weight compared to the Ctrl group (Figure 1A), with significant differences in the body growing slopes (Control = 0.9821 g/week vs. 2.203 g/week in the HC group, *p* = 0.0064), greater body weight gain (final weight − initial weight) (Figure 1B), and Lee index values above 310, corresponding to obesity (Table 1). Additionally, biochemical parameters (glucose, total cholesterol, triglycerides, and HDL) were evaluated. Only triglyceride levels increase, reaching values twice those in the control group (Table 1).

In addition, we calculated the triglyceride/HDL-C ratio, an indirect marker of insulin resistance, that has been associated with increased visceral and hepatic fat in obese individuals [11]. The HC group also showed a significant increase (>2) in the TG/HDL-C ratio. In contrast, the Ctrl group with normal weight showed values <2, indicating a low risk. Together, these results confirm the presence of obesity and altered metabolism in animals fed the HC diet.

Furthermore, muscle function was evaluated to assess the impact of obesity on the sarcopenia or skeletal muscle dysfunction-associated decrease in mobility. The HC diet group showed a 37% decrease in treadmill standing time (Figure 1C) and a 27% decrease in grip strength (Figure 1D) compared to the Ctrl group. Based on these results, we consider that the obesogenic/sarcopenic model was successfully developed. Hereafter, we will refer to this group as “Obese” (OB).

Effect of (−)-epicatechin treatment on skeletal muscle dysfunction

**Figure 2:**
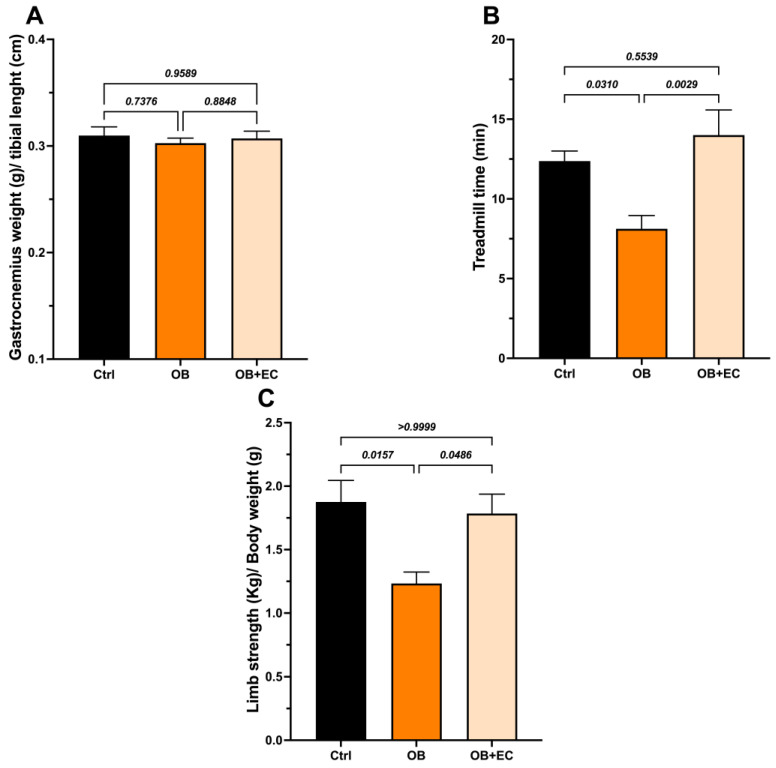
Effect of EC treatment of obese (OB) rats on (**A**) the weight of the gastrocnemius; (**B**) the treadmill time, and (**C**) the Limb strength. Data are shown as mean ± SEM. Ctrl (*n* = 8), OB (*n* = 8), and OB + EC (*n* = 8).

The wet weight of the gastrocnemius muscle did not differ between the obesity model (OB) and control rats. We also did not find significant differences with the EC treatment (Figure 2A).

Interestingly, although no changes in muscle mass were observed, obese animals had shorter average walking times than controls, and treatment with EC significantly improved muscle function. The OB + EC group spent 72% more time on the treadmill than the OB group (Figure 2B) and performed as well as the normal-weight (control) group.

Regarding grip strength, a decrease in strength was observed in the OB group and a significant recovery in the OB + EC group (Figure 2C).

Effect of (−)-epicatechin on mitochondrial DNA content

**Figure 3:**
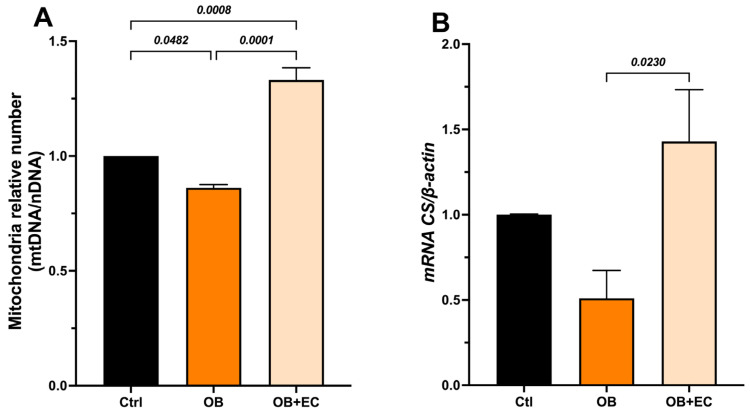
(**A**) Effect of EC on mitochondrial biogenesis, and (**B**) indirect activity measured as expression of citrate synthase (Cs) in gastrocnemius muscle. Data are shown as mean ± SEM. Ctrl (*n* = 8), OB (*n* = 8), and OB + EC (*n* = 8).

To determine whether the observed effects on muscle function were due to changes in mtDNA content (mitochondrial number), we quantified a mitochondrial gene (*Nrn2*) and a nuclear gene (*Gapdh*), and used the mtDNA/nDNA ratio as a proxy for relative changes in mitochondrial number. In the gastrocnemius muscle, we found that mitochondrial content was significantly lower in the OB group compared to the control group (14%), while a significant increase was observed in the OB + EC group, 54% higher than the untreated group (OB) and even higher (33%) than the mitochondrial content of the normal-weight control group (Figure 3A).

Mitochondrial activity was indirectly evaluated through the expression of citrate synthase (Cs), which was significantly increased in the OB + EC group compared to the OB group (Figure 3B).

On the other hand, the expression of genes related to mitochondrial processes: biogenesis (*Pgc1-α*, *Tfam*), fusion (*Mfn1* and *Opa1*), fission (*Drp1* and *Fis1*), and mitophagy (*Pink1* and *Pkn*) was evaluated to determine whether these processes were related to the observed changes in relative mtDNA content.

**Figure 4:**
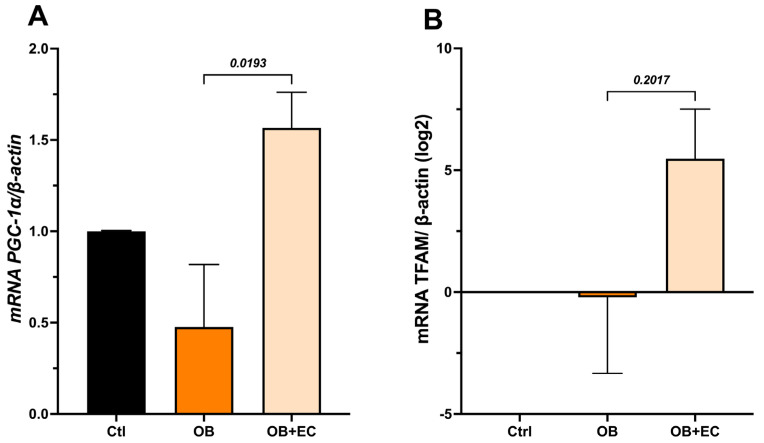
Effect of EC on mitochondrial biogenesis-associated inducers: (**A**) *Pgc-1a* and (**B**) *Tfam*, in gastrocnemius muscle. Data are shown as mean ± SEM.

The results showed that the OB group exhibited a significant decrease in *Pgc-1α* expression; conversely, the OB + EC group showed an increase in expression, reaching levels comparable to those of the Ctrl group (Figure 4A). Furthermore, *Tfam* expression, which may be highly variable, showed no statistically significant differences. Interestingly, the Log2 analysis of change values relative to controls shows a relative increase in the OB + EC group, although this difference was not statistically significant (Figure 4B).

**Figure 5:**
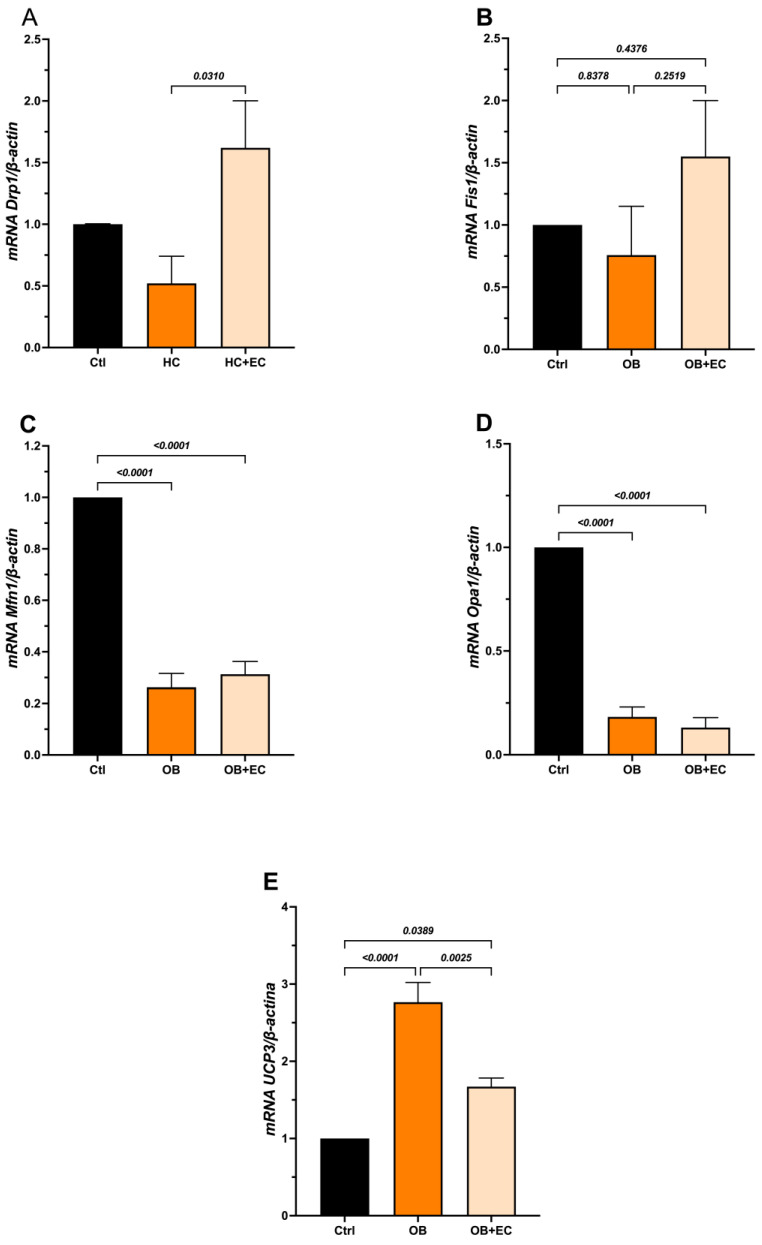
Effect of EC on mitochondrial dynamics; fission (**A**,**B**), fusion (**C**,**D**), and functional adaptation (**E**) in gastrocnemius. Data show mean ± SEM. Ctrl (*n* = 8), OB (*n* = 8), and OB + EC (*n* = 8).

On the other hand, in the mitochondrial dynamics analysis, we examined the expression of *Drp1* and *Fis1.* The results showed that OB induces a slight decrease in *Drp1*. Interestingly, the OB + EC group showed a significant increase in *Drp1* expression compared to the OB group (Figure 5A). However, *Fis1* expression did not change between groups in the OB group, and in the OB + EC group showed a nonsignificant increase (Figure 5B).

Regarding the fusion process, both *Mfn1* and *Opa1* expression decreased significantly in the OB and OB + EC groups compared with the Ctrl group, suggesting that obesity negatively affects this process, although EC treatment did not reverse or attenuate this effect (Figure 5C,D).

To evaluate mitophagy, *Pink1* and Parkin (*Pkn*) expression was assessed; however, both markers showed undetectable levels under the experimental conditions). Additionally, we evaluated *Ucp3* expression as an indicator of mitochondrial functional adaptation. Our results showed that the OB group had increased expression, whereas the OB + EC group had decreased expression compared to the OB group (Figure 5E).

Table 2 summarizes the changes in gene expression observed:

## 3. Discussion

The main findings of this work were: a significant decrease in mobility and strength in obese female rats, mediated by reduced mitochondrial numbers, activity, and dynamics, but not by changes in muscle size or weight. Interestingly, treatment with the flavanol EC induced mitochondrial biogenesis and altered mitochondrial dynamics (fission and fusion) and activity, as measured indirectly by changes in citrate synthase and *Ucp3* expression.

Currently, the increase in obesity, particularly in women, has led to a significant increase in dysfunctional conditions, including diminished mobility and sarcopenia. Given that physical and other conditions prevent women from initiating or maintaining physical activity, it is of great interest to develop practical approaches to attenuate or reverse the progression of these conditions and improve the quality of life for this population.

In the present study, we showed that administration of a high-fat, high-carbohydrate diet induced an obese phenotype, accompanied by alterations in skeletal muscle function.

Several studies in animals and humans have shown that EC induces beneficial effects on the skeletal muscle and the cardiovascular system, reducing risk factors such as arterial hypertension, endothelial dysfunction, damage to skeletal muscle structure, and mitochondrial malfunction by promoting mitochondrial biogenesis, with no adverse effects reported [9,12].

Our results showed that treatment of obese rats with EC increased (doubled) the time spent on the treadmill walking test, demonstrating a significant improvement in physical performance. This finding is consistent with previous reports describing similar effects of exercise training and EC on muscular endurance [12,13]. It is noteworthy that this benefit was maintained despite the high-calorie diet being continued until the end of the experimental protocol, highlighting EC’s potential to counteract the adverse effects partially of a metabolically unfavorable condition.

EC has been proposed as a possible “exercise mimetic” by having pleiotropic effects on skeletal muscle and the heart, such as increased muscle capillarity, which increases the potential for oxygen flow to the muscles, increased mitochondrial biogenesis, and increased oxidative capacity [12].

In this group of female rats, EC did not significantly improve grip strength compared with controls, as has been shown in males [12]. This level discrepancy could be attributed to the fact that the reported effects of EC in females predominantly focus on processes other than protein synthesis, which has been reported to be closely linked to increased grip strength in males [12,14]. This possibility is reinforced by the absence of significant differences in the muscle weights; however, EC treatment induced a significant recovery of the obesity-induced decrease in strength.

Since mitochondrial dysfunction is a major mechanism in the development of sarcopenia [15], we evaluated mitochondrial DNA (mtDNA) content as an indirect marker of mitochondrial dysfunction. Our results showed a significant decrease in relative mtDNA content in the gastrocnemius muscle of the OB group. However, treatment with EC improved these levels, which were even higher than in the control group, suggesting a compensatory effect that promotes mitochondrial biogenesis. These results are consistent with reports that a high-fat, high-sucrose diet reduces mitochondrial DNA content in skeletal muscle. Prolonged hyperglycemia and hyperlipidemia promote the accumulation of energy substrates in muscle, thereby increasing intramyocellular lipids, generating reactive oxygen species (ROS), and ultimately leading to mitochondrial dysfunction [16,17,18].

To determine whether the observed variations in mitochondrial DNA content are due to EC’s effect on mitochondrial biogenesis, the expression of *Pgc-1α* and *Tfam*, key regulators of this process, was evaluated. EC treatment increased *Pgc-1α* levels compared with the untreated obese group. Regarding *Tfam*, although a similar increase to that observed for *Pgc-1α* was observed, it was not statistically significant. The results obtained are consistent with reports suggesting that EC can induce mitochondrial biogenesis by binding to the G protein-coupled estrogen receptor (GPER), which activates Nrf2, Tfam, and Pgc-1α, thereby improving muscle endurance and reducing fatigue [11,19,20].

On the other hand, the expression of *Drp1* and *Fis1* as mitochondrial fission markers, and *Mfn1* and *Opa1* as fusion markers, showed that EC administration significantly increased *Drp1* expression compared to the other groups, while *Fis1* did not show significant differences between groups, and both fusion markers, *Mfn1* and *Opa1*, showed decreased expression in the OB and OB+CE groups as compared with the control group, a pattern previously described in conditions of obesity and consumption of HC diets [21,22].

*Drp1* is the central mediator of mitochondrial fission, enveloping and constricting mitochondrial tubules to facilitate mitochondrial division [23]. Experimental evidence has shown that inhibition of *Drp1* in mouse skeletal muscle for 4 months causes severe muscle atrophy [24]. Similarly, loss of this protein results in enlarged and dysfunctional mitochondria, resulting in muscle wasting and weakness [25].

Although no previous studies have directly evaluated the effect of EC on mitochondrial fission in skeletal muscle, reports indicate that this flavanol can increase levels of mitofilin and porin, mitochondrial proteins involved in mitochondrial remodeling [11,26].

Previous studies have shown that the simultaneous absence of *Mfn1* and *Mfn2* in murine skeletal muscle causes severe mitochondrial dysfunction, mitochondrial DNA damage, and growth inhibition. The deletion of both proteins resulted in a significant decrease in physical performance [27,28,29]. Furthermore, deregulation of *Mfn1* and *Mfn2* expression inhibits mitochondrial fusion, leading to an increase in “broken” mitochondria and a decrease in mitophagy^7^. Regarding *Opa1*, reports indicate that its reduction leads to mitochondrial fragmentation, which, although it is mostly a pathological phenomenon that contributes to cellular dysfunction, may also play a role in the elimination of damaged mitochondria as a possible quality-control mechanism [30].

Mitophagy is a process mediated mainly by the *Pink1*-Parkin pathway, which allows the selective elimination of dysfunctional or excess mitochondria as part of a quality control mechanism to prevent the accumulation of mitochondrial damage [31]. In our study, however, *Pink1* and *Pkn* were undetectable. It has been documented that conditions such as neurodegenerative diseases, inflammation, cancer, and metabolic disorders can be associated with impaired or suppressed mitophagy [32], which could partly explain the absence of *Pink1* and Parkin detection in our study.

Furthermore, we evaluated citrate synthase expression, a key Krebs cycle enzyme used as a marker of mitochondrial metabolic activity [33]. Several studies have shown that increased expression is reflected in greater mitochondrial density, oxidative capacity, and improved physical performance [34,35].

Our results showed that EC increased the expression levels of this enzyme in the gastrocnemius muscle. These results suggest that EC can enhance mitochondrial oxidative activity. This effect is also associated with improved physical endurance in the EC-treated group, as evidenced by greater performance in the treadmill test, as fatigue resistance largely depends on efficient oxidative energy metabolism. It has been reported that EC can stimulate CS activity in C2C12 myotubes, accompanied by increased mitochondrial biogenesis, which is partially mediated by the GPER receptor. A similar effect has been observed in an in vivo model, in which EC treatment in senile mice restored Cs activity in most organs tested, including skeletal muscle [36].

The gastrocnemius muscle is composed primarily of type II fibers with lower mitochondrial density and greater dependence on glycolytic metabolism, making it prone to fatigue. However, in our study, animals treated with EC showed greater fatigue resistance, reflected in longer treadmill stays. This finding suggests a possible adaptation toward a more oxidative metabolic profile in the gastrocnemius, potentially associated with changes in the proportions or functions of muscle fibers. This hypothesis is supported by the increase in mitochondrial biogenesis markers and Cs activity observed with EC, indicating an improvement in oxidative capacity. This is supported by studies showing that overexpression of Pgc-1α increases mitochondrial DNA content and promotes the conversion of glycolytic to oxidative muscle fibers [37].

Furthermore, the expression of *Ucp3*, an oxidative phosphorylation uncoupling protein that decreases the proton gradient and thereby reduces ROS production, was evaluated. This protein is primarily present in skeletal muscle [38]. Studies conducted in *Ucp3*-deficient mice (*Ucp3*-/-) showed elevated ROS levels, suggesting a protective role for Ucp3 against oxidative stress [39].

In our study, EC decreased *Ucp3* expression levels compared to the untreated obese group. The increased Ucp3 expression in animals on a high-calorie diet suggests an adaptive response to the metabolic and oxidative stress associated with obesity, likely aimed at limiting ROS production. However, the decreased *Ucp3* expression observed in EC-treated animals can be interpreted as an indication of restored mitochondrial function, in which reduced oxidative stress precludes the activation of compensatory mechanisms, such as *Ucp3* overexpression.

Finally, by integrating the results, we suggest that EC promotes mitochondrial renewal by increasing mitochondrial biogenesis and eliminating damaged mitochondria through increased mitochondrial fission. Although we were unable to evaluate mitophagy marker expression in our study, this process likely occurs through other pathways or later. Furthermore, EC increases functional mitochondria via CS expression and protects against oxidative damage caused by the metabolic stress to which the animals are subjected, as reflected in the decrease in *Ucp3*. Together, these molecular effects were associated with improved physical performance in treated animals.

These findings reinforce the potential of EC as a modulator of mitochondrial function in conditions of metabolic dysfunction associated with obesity, thereby attenuating the mechanisms underlying sarcopenia. Altogether, the results reported in this work in a group of female obese rats demonstrate a positive effect of EC improving the diminished mobility and mitochondria levels and dynamics that is independent of muscle hypertrophy setting the base to more profound studies leading to the implementation of EC use as a supplementary maneuver link to changes in diet together with moderate exercise to improve mobility and QoL in O women.

This work has some limitations: we did not observe a decrease in muscle mass, as in other previously reported models; we believe this may be related to gender differences in EC-induced effects. Another limitation is the single dose used in the study; however, several studies by us and others using the dose have shown positive EC effects. More work is necessary to fulfill these gaps.

## 4. Materials and Methods

### 4.1. Study Design and Animal Model

This study was approved by the institutional committee (# 2018-1-160) with approval date 12 July 2018 and followed the guidelines of Mexican Official Standard NOM-062-ZOO-1999 [40]; Institutional research and ethics committees approved the protocol: Procedures and technical specifications for the production and animal care complied with the recommendations of Guide for the Care and Use of Laboratory Animals of the National Institutes of Health (Institute of Laboratory Animal Resource (US). Committee on Care and Use of Laboratory Animal 2011).

Twenty-four 6-month-old female Wistar rats were used. The rats were initially randomly separated into two groups: (1) a control group (Ctrl) (*n* = 8) receiving standard Purina Nutricube food and water ad libitum; and (2) a high-fat, high-sugar (HC) diet group (*n* = 16) (56% standard food, 20% condensed milk, 12% standard sugar, and 12% edible fat from bovine tallow) plus water supplemented with 30% standard sugar ad libitum. The animals were fed the HC diet for 15 weeks to induce obesity, as evidenced by increases in body weight, Lee index, and lipid profile alterations, and by abdominal fat accumulation, and to promote a sarcopenic phenotype (decreases in muscle mass, strength, and physical performance). Weight and body measurements (naso-anal distance and abdominal circumference) were recorded weekly during the 15-week obesity-induction period. Functional tests of grip strength and mobility (treadmill) were performed at the end of the diet induction period and after treatment. Blood samples were collected from the lateral tail vein to determine glucose concentrations and lipid profiles (total cholesterol, triglycerides, and HDL cholesterol) at the end of the diet. To determine whether the animal model exhibited an obese phenotype, the Lee index (body weight ^1/3^/naso-anal distance), a commonly used parameter for assessing obesity in rats, was calculated [41,42]. A Lee index value greater than 310 is considered indicative of obesity. After 15 weeks on the HC diet and once the obesity phenotype was established, the animals were randomly divided into the following groups: (1) obese animals plus vehicle (water by gavage) (OB) (*n* = 8) and (2) obese animals plus 1 mg/kg/day of (−)-epicatechin (Sigma-Aldrich (Saint Louis, MO, USA), E1753-1G) in water (OB + EC by gavage) (*n* = 8) during 4 weeks, the control group was maintained under standard diet + vehicle by gavage.

We chose this treatment period and single dose based on prior reports suggesting that positive, significant effects are observed within 15 days of EC treatment [12,13], even when EC bioavailability is only around 30–40% [43].

### 4.2. Functional Tests

Muscle strength. Forelimb strength was assessed with a grip strength meter [44].

Treadmill. Rats were familiarized with the treadmill at a minimum speed (~12 m/min) for 5 min over 2 days before testing. The test consisted of rats walking on a treadmill at a minimum speed until they could no longer walk. A compressed air gun was used at the rear of the treadmill to discourage the animals from stopping. The test ended when the animals could no longer maintain their normal walking position, and the total walking time was recorded [44].

### 4.3. Mitochondrial DNA (mtDNA) Content

After the experimental period, animals were euthanized using sodium pentobarbital, and 10 mg of gastrocnemius tissue were disaggregated with proteinase K for 24 h at 55 °C. Total DNA was extracted using the GeneJet Whole Blood Genomic DNA Purification Kit (ThermoScientific, Waltham, MA, USA) according to the manufacturer’s instructions. Relative mtDNA quantification was performed using the qPCRBIO SyGreen^®^ Blue Mix Lo-ROX kit (PCR BIOSYSTEMS, London, UK). Amplification conditions were 95 °C/2 min followed by 40 cycles (95 °C/5 s, 60 °C/20 s). The relative mtDNA copy number was estimated from the mtDNA/nDNA ratio using the mitochondrial 16S ribosomal RNA gene (*Rnr2*) and glyceraldehyde-3-phosphate dehydrogenase (*Gapdh*) as a nuclear reference. ΔCt was calculated as Ct(mtDNA, RNR2) − Ct(nDNA, GAPDH), and relative mtDNA content was expressed as 2^−ΔΔCt^ normalized to the Ctrl group mean. The oligonucleotide sequences used are shown in Table 3.

### 4.4. Gene Expression Quantification by RT-qPCR

Total RNA was extracted from gastrocnemius muscles using the Direct-zol™ RNA MiniPrep Plus Kit (ZYMO RESEARCH, Irvine, CA, USA) according to the manufacturer’s instructions. cDNA was synthesized using the Accuris qMax™ First Strand cDNA Synthesis Flex Kit (Innovative Research, Tokio, Japan). qPCR was performed on a real-time PCR system (Applied Biosystems, Waltham, MA, USA) using the qPCRBIO SyGreen^®^ Blue Mix Lo-ROX Kit (PCR-BIOSYSTEMS). Amplification conditions were 95 °C/2 min followed by 40 cycles (95 °C/5 s, 60 °C/20 s). The oligonucleotide sequences used are shown in Table 3.

### 4.5. Statistical Analysis

All results are presented as mean ± standard error of the mean (SEM). The Shapiro–Wilk test was used to assess normality. An unpaired t-test (normal data) or a Mann–Whitney test (non-parametric data) was used to assess differences between group means, and ANOVA with multiple comparisons followed by Tukey’s test (normal data) or Kruskal–Wallis test (non-parametric data was used when applicable; *p*-values < 0.05 were considered statistically significant. Linear regression with analysis of slope differences was performed. All analyses were performed using GraphPad Prism version 10.0.

## Figures and Tables

**Table 1:** Effect of diet and aging on anthropometric and biochemical parameters.

Parameters	Control	HC	*p*-Value
Weight gain (g)	10.02 ± 4.49	33.68 ± 6.41	0.0240
Lee index	301.1 ± 2.20	314.60 ± 2.20	0.0005
Glucose (mg/dL)	64.68 ± 5.05	69.87 ± 4.70	ns
Triglycerides (mg/dL)	70.18 ± 29.63	153.10 ± 67.06	<0.0001
Total cholesterol (mg/dL)	58.45 ± 7.51	63.79 ± 3.99	ns
HDL-cholesterol (mg/dL)	37.82 ± 2.47	39.79 ± 1.80	ns
Triglycerides/HDL-c ratio (mg/dL)	1.89 ± 0.17	4.10 ± 0.48	<0.0001

**Table 2:** Summary of gene expression in the gastrocnemius muscle.

Process	Gen	Ctrl	OB	OB + EC
Biogenesis	*Pgc-1α*	↔	↓	↑
	*Tfam*	↔	NS	NS
Fission	*Drp1*	↔	↓	↑
	*Fis1*	↔	NS	NS
Fusion	*Mfn1*	↔	↓	↓
	*Opa1*	↔	↓	↓
Activity	*Cs*	↔	↓	↑
	*Ucp3*	↔	↑	↓
Mitophagy	*Pink1*	↔	Undetectable	Undetectable
	*Pkn*	↔	Undetectable	Undetectable

**Table 3:** Oligo sequences used were as follows in the different analysis are.

m-RNA Sequences	Forward (5′ to 3′)	Reverse (5′ to 3′)
*Pgc1-a*	TAT GGA GTG ACA TAG AGT GTG CT	CCA CTT CAA TCC ACC CAG AAA G
*Tfam*	GGA ATG TGG AGC GTG CTA AAA	ACA AGA CTG ATA GAC GAG GGG
*Drp1*	AGA GCA CGC AAT TTG AAT ATG CC	ATA GTC CCG CTG TTC CTC TTT
*Mfn1*	CCT ACT GCT CCT TCT AAC CCA	AGG GAC GCC AAT CCT GTG A
*Fis1*	TGT CCA AGA GCA CGC AAT TTG	CCT CGC ACA TAC TTT AGA GCC TT
*Cs*	GGA CAA TTT TCC AAC CAA TCT GC	TCG GTT CAT TCC CTC TGC ATA
*Opa1*	ACA GCA AAT TCA AGA GCA CGA	TTG CGC TTC TGT TGG GCA T
*Ucp3*	CCG ATT TCA AGC CAT GAT ACG C	CCT GGC GAT GGT TCT GTA GG
*Actb*	CCG CGA GTA CAA CCT TCT TG	GCA GCG ATA TCG TCA TCC AT
DNA sequences	Forward (5′ to 3′)	Reverse (5′ to 3′)
*Rnr2*	AGC TAT TAA TGG TTC GTT TGT	AGG AGG CTC CAT TTC TCT TGT
*Gapdh*	GGA AAG ACA GGT GTT TTG CA	AGG TCA GAG TGA GCA GGA CA

## Data Availability

Data is contained within the article.
